# Serological determinants of COVID-19

**DOI:** 10.1186/s13062-020-00276-1

**Published:** 2020-11-02

**Authors:** Annalisa Noce, Maria Luisa Santoro, Giulia Marrone, Cartesio D’Agostini, Ivano Amelio, Andrea Duggento, Manfredi Tesauro, Nicola Di Daniele

**Affiliations:** 1 UOC of Internal Medicine-Center of Hypertension and Nephrology Unit, Department of Systems Medicine, University of Rome Tor Vergata, via Montpellier 1, 00133 Rome, Italy; 2 Laboratory Pathologist Director of Artemisia Lab – Alessandria, Via Piave, 76 00187 Rome, Italy; 3 PhD School of Applied Medical, Surgical Sciences, University of Rome Tor Vergata, via Montpellier 1, 00133 Rome, Italy; 4 Department of Experimental Medicine, University of Rome Tor Vergata, via Montpellier 1, 00133 Rome, Italy; 5 Laboratory of Clinical Microbiology, Policlinico Tor Vergata, viale Oxford 81, 00133 Rome, Italy; 6 School of Life Sciences, University of Nottingham, Nottingham, UK; 7 Department of Biomedicine and Prevention, University of Rome Tor Vergata, via Montpellier 1, 00133 Rome, Italy

**Keywords:** SARS-CoV-2, COVID-19, Point of care, Lateral flow immunoassay, Automated chemiluminescent immunoassay, Serological tests, Laboratory detection

## Abstract

**Background:**

Severe acute respiratory syndrome coronavirus 2 (SARS-CoV-2) infection spreaded rapidly worldwide, as far as it has become a global pandemic. Therefore, the introduction of serological tests for determination of IgM and IgG antibodies has become the main diagnostic tool, useful for tracking the spread of the virus and for consequently allowing its containment. In our study we compared point of care test (POCT) lateral flow immunoassay (FIA) vs automated chemiluminescent immunoassay (CLIA), in order to assess their specificity and sensibility for COVID-19 antibodies detection.

**Results:**

We find that different specificities and sensitivities for IgM and IgG tests. Notably IgM POCT FIA method vs CLIA method (gold standard) has a low sensitivity (0.526), while IgG POCT FIA method vs CLIA method (gold standard) test has a much higher sensitivity (0.937); further, with respect of IgG, FIA and CLIA could arguably provide equivalent information.

**Conclusions:**

FIA method could be helpful in assessing in short time, the possible contagiousness of subjects that for work reasons cannot guarantee “social distancing”.

## Background

Coronavirus disease 2019 (COVID-19) is a novel coronavirus pneumonia caused by severe acute respiratory syndrome coronavirus 2 (SARS-CoV-2) [1, 2]. Emergence of new infectious diseases poses serious clinical issues [3–9], this new infection was first encountered in December 2019 in Wuhan, Hubei Province, China, and then spread worldwide taking on the appearance of health emergency of international concern. Starting from February 2020, the COVID-19 outbreak spread in Europe, particularly affecting northern Italy and Spain [10–12]. World Health Organization (WHO), on 11th March 2020 declared COVID-19 disease a global world pandemic.

SARS-COV-2 belongs to the beta coronavirus family along with other human pathogens known as SARS-CoV and Middle East respiratory syndrome coronavirus (MERS-Cov) [13]. As COVID-19 was identified as a health emergency by WHO, large-scale population testing proved to be of crucially important to identify and isolate symptomatic and asymptomatic case, in the global efforts to contain its expansion.

**Fig. 1:**
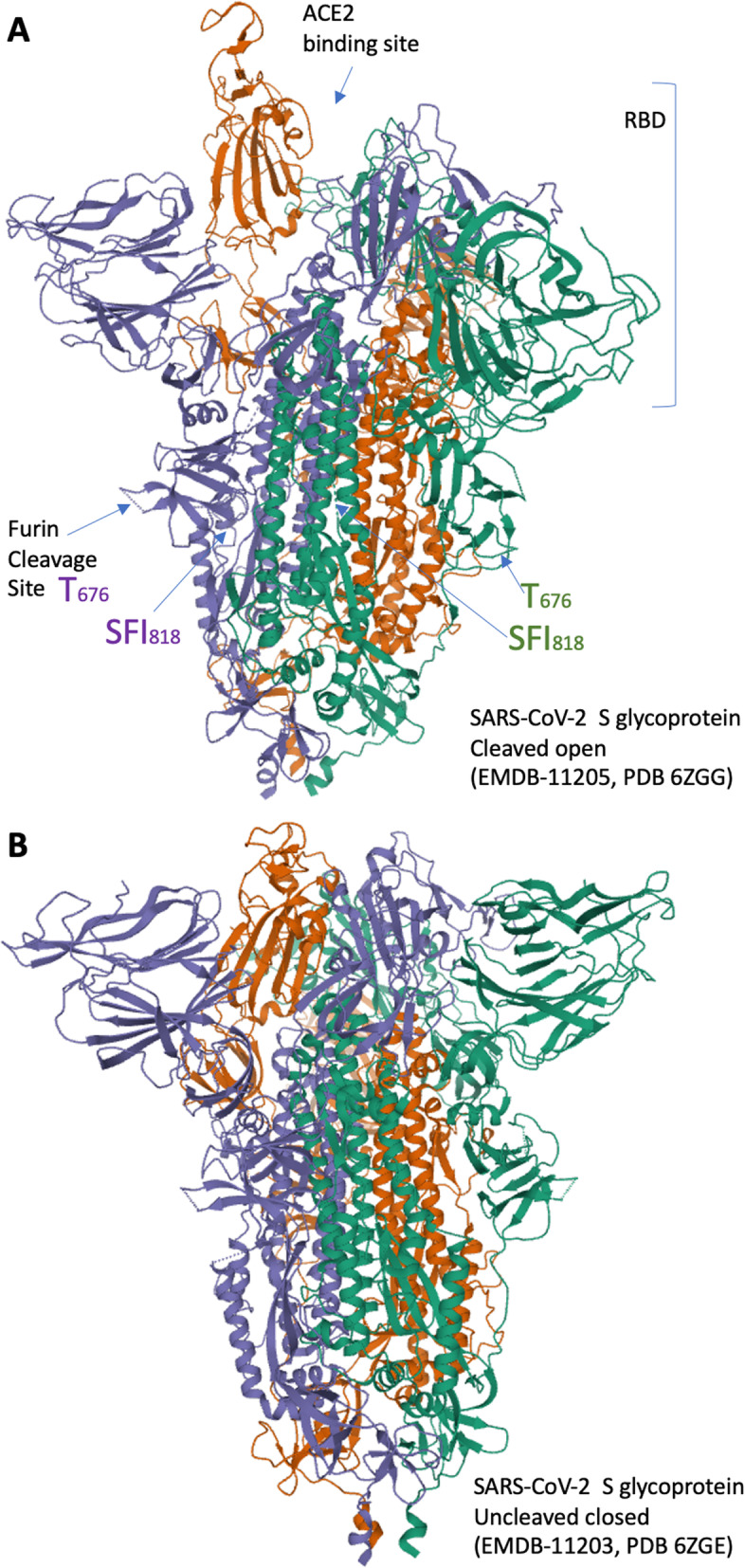
Spike protein of the SARS-CoV-2. **a**, **b** 3D structure of the Spike protein in the cleaved (**a**) or uncleaved (**b**) conformations (EMDB-11205, PDB 6ZGG or EMDB-11203, PDB 6ZGE respectively). Panel “a” also indicates Furin cleavage site

In December 2019, SARS-COV-2 was firstly transmitted to humans through human-animal contact at live animals market in Wuhan (China) [14]. SARS-CoV-2 belongs to the subfamily of the Coronavirinae, which is part of the order Nidoviralescoronaviruses. It is a single-stranded RNA-enveloped virus, containing 4 structural proteins (from the 3’end open reading frames- ORF) and 16 accessory proteins (nsp 1 to nsp 16) from the 5’end ORFs. The viral envelop contains structural proteins E and M, while the N protein nucleocapsid binds the viral RNA. The S glycoprotein is the key player for the interaction with angiotensin-converting enzyme 2 (ACE2) on the host cells (Fig. 1) [15]. The interaction between ACE2 and the S glycoprotein was conserved also in the SARS-CoV, the virus responsible of the SARS outbreak of 2002–2003. The S protein binds to the receptor to target host organism cells. The virus uses also other host cell receptors such as the type 2 transmembrane serine protease (TMPRSS2), to trigger the endocytotic process employed to access the cells [16]. Viral polyproteins are expressed in the host cell, RNA can be synthetized via its RNA-dependent RNA polymerase and new viral particles can be produced and released.

**Fig. 2:**
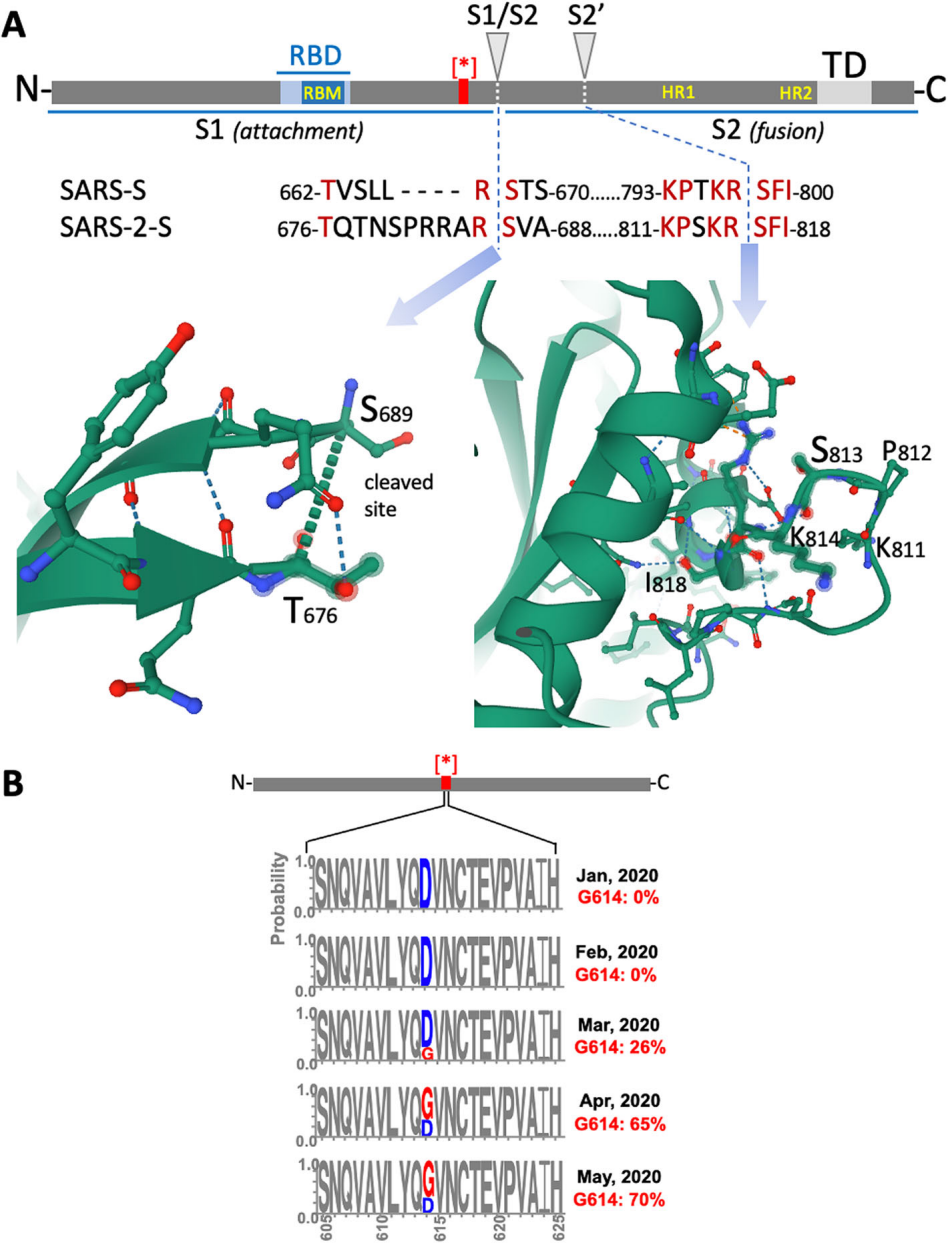
Structure and domain organization of the Spike protein of the SARS-CoV-2. **a** The S1 subunit includes the RBD, which is responsible for the interaction with the ACE2 receptor on the host cell membrane. The subunit S2 includes the membrane fusion complex (fusion peptide, heptad repeats HR 1 and HR2), anchors the S2 subunits to the viral membrane with its transmembrane domain, and interacts with the viral ribonucleoprotein complex through its endodomain. **b** D614G mutation in the Spike protein and frequency across the time

Cleavage at the S1/S2 and the S2’ site of the S protein by the proteases of the host cell is necessary for membrane fusion [17] (Fig. 2). Cleaved S protein is therefore the activated form ready to enter the cell. This proteolytic step can also occur in the constitutive secretory pathway of infected cells by endosomal cathepsins B and L and furin [18]. Here, the viral membrane the S protein can be cleaved (primed) in two segments (Fig. 2). The N-terminal S1 segment is responsible for the interaction with the host cell receptor, as it contains a signal peptide and the receptor binding domain (RBD). The S2 segment anchors the S protein to the viral membrane, contains the fusion peptide which mediates the fusion of the viral membrane with the plasma membrane of the target cell. The proteases responsible for the S protein activation represent promising drug targets for the treatment of the disease, following failure of first attempts, such as hydroxychloroquine [19].

Many mutations in the SARS-CoV-2 virus have been observed. One among the most prevalent is the D614G, at the C terminal region of subunit S1 of the Spike protein, which is the region in which subunit S1 associates with S2 (Fig. 2b). How and from where this mutation emerged is not clear, however it appears to give the virus a decisive transmission advantage over the non-mutated variant [20].

SARS-COV-2 infection displays a broad spectrum of symptoms ranging from asymptomatic forms, mild to moderate symptoms, up to severe respiratory symptoms and lung abnormalities which require intensive care including assisted oxygenation [10, 21]. The most frequently symptoms are: fever, dry cough, upper tract respiratory symptoms, myalgia, anosmia, ageusia and headache [22, 23]. Other fearsome complications are represented by acute respiratory distress syndrome (ARDS), respiratory failure and liver injury, acute myocardial injury and acute kidney injury, septic shock and multiple organ failure [24]. Recently, the alteration of the intestinal microbiota has been described in patients with COVID-19, as occurs in chronic non-communicable diseases (CNCDs) [25, 26]. In future, the possible understanding of the mechanisms underlying the alterations of the intestinal microbiota following SARS-CoV-2 infection could represent a new diagnostic biomarker and therapeutic target for the fight against COVID-19. The incubation period of the infection ranges from 0 to 24 days [27].

Despite SARS-COV-2 infection mainly affects the geriatric population (subjects aged over 65 years) and individuals with altered immune system or with chronic diseases (such arterial hypertension, chronic kidney disease, chronic obstructive bronchopathy, etc.), in the last months it has been observed a greater spread of the virus in younger subjects due to unappropriated social behavior (disregarding social distancing recommendation) [28–30].

Serological tests, for the determination of IgG and IgM are one of the most important components of the public health response to COVID-19, along with viral diagnostic tests, for the contact tracing and regulation of lockdown measures. However, given the simplicity of the method of serological tests, especially those performed through a point of care test (POCT) method, able to detect simultaneously the presence of IgM and IgG, their use could probably reduce the extent of the shielding required to obtain a better reduction of COVID-19 transmission, in order to allow a considerable number of individuals to return to social and economic interactions [31].

Accurate and rapid diagnostic tests will be critical for achieving control of COVID-19. Omics approaches and data integration have facilitated identification of biomarkers for many diseases [32–36]. Similarly, production models have been proven as useful tools [37, 38], however serology represents a critical step in the COVID-19 control. Diagnostic tests for COVID-19 fall into two main categories: molecular tests that detect viral RNA, and serological tests that detect anti-SARS-CoV-2 immunoglobulins. Reverse transcriptase polymerase chain reaction (RT-PCR), a molecular test, is widely used as standard for diagnosis of COVID-19; however, its limitations include false negative results [39, 40] that affect diagnostic accuracy over the disease course [41], and precarious availability of test materials [42]. Serological tests have generated substantial interest as represent an alternative or complementary approach to RT-PCR in the diagnosis of acute infection, as recently reported by Long and colleagues [43, 44]. The authors of this study showed that SARS-CoV-2 immunoglobulins, tested in 285 subjects, were present in 100% of case within 19 days of symptoms onset. Hence, they concluded that serological tests represent a useful tool, for the diagnosis of suspected cases with negative RT-PCR and for the diagnosis of asymptomatic subjects. Serological tests might be cheaper and easier to implement in the POCT. A clear advantage of these tests over RT-PCR is that they can identify individuals previously infected by SARS-CoV-2, even if they never underwent testing while acutely ill. Serological tests could be deployed as surveillance tools to better understand the epidemiology of SARS-CoV-2 and potentially inform individual risk of future disease. Many serological tests for COVID-19 have become available in a short period, including some marketed for use as rapid (POCT).

In this study is to compare two different diagnostic laboratory methods, rapid lateral flow immunoassay (FIA) vs automated chemiluminescent immunoassay (CLIA) to assess their specificity and sensibility against COVID-19 antibodies detection. In the evaluation of COVID-19 positivity and assessment of its diffusion, it should be advised to develop a rapid laboratory test for its serological early-diagnosis.

## Results

Table 1 shows the confusion matrix for the IgM tests, while Table 2 shows the confusion matrix for the IgG tests. Table 3 presents the statistics summary. As it can been seen, the specificity of both COVID-19 IgM FIA and COVID-19 IgG CLIA tests were 1, i.e. no false positive results were recorded for neither of the two tests. Conversely a difference in terms of sensitivity was identified the IgM and IgG tests: while the COVID-19 IgM FIA test registered a sensitivity as low as 0.526 (high ratio of false negative results), the COVID-19 IgG FIA test displayed a much higher sensitivity equal to 0.937. The overall accuracy was also significantly different: 0.878 (CI: 0.782–0.943) vs 0.973 (CI: 0.906–0.997) for IgM and IgG respectively.

**Table 1:** Confusion matrix for the classes relative to IgM detection

IgM classes		Covid-19 CLIA	
		Negative	Positive
**Covid-19 FIA**	Negative	55	9
	Positive	0	10

**Table 2:** Confusion matrix for the classes relative to IgG detection

IgG classes		Covid-19 CLIA	
		Negative	Positive
**Covid-19 FIA**	Negative	42	2
	Positive	0	30

**Table 3:** Summary table of the statistical measures for FIA vs CLIA test

Statistics	IgM (FIA vs CLIA)	IgG (FIA vs CLIA)
Accuracy	0.8784	0.973
Accuracy 95% CI	(0.7816, 0.9429)	(0.9058, 0.9967)
McNemar’s test p-value	0.007661	0.4795
Sensitivity	0.5263	0.9375
Specificity	1	1

The McNemar test *p*-values were also very different from IgM and IgG tests. In relation to the IgM, the highly significant McNemar test *p*-value = 0.00076 indicates that the FIA and CLIA tests convey different information and are not interchangeable, with a very high significance. In the IgG, the McNemar test *p*-value = 0.48 indicates that we cannot reject the hypothesis that the FIA and CLIA tests are statistically equivalent.

With the FIA method, no significant differences were observed between results obtained from capillary blood tests and results obtained from venous blood test.

## Discussion

This study aims to assess whether a POCT could be able to correctly screen IgM or IgG antibodies against SARS-CoV2. We tested an analytical method (FIA method) of rapid detection of IgM/ IgG antibodies which was compared with a gold-standard method (CLIA method). The antibody response follows the spread of the pathogen in the host and it is characterized by the production and secretion of antibodies from B lymphocytes (adaptive immune system) [45]. IgM are the first antibody response against pathogens, subsequently IgG are produced and also represent the immunological memory.

Recently, many commercial rapid tests (among these POCT FIA) have been developed and CE-marked [46]. The results of many studies showed that their global sensitivity, specificity, positive predictive value (PPV) and negative predictive value (NPV) were equivalent to the ELISA IgG/IgM or the CLIA IgG/IgM tests [47]. Similarly to previous studies, we found accordance between the two analytical methods. The results showed a good sensitivity (88.6%) and specificity (90.6%) with the rapid test. Moreover, we obtained similar results on both venous blood and capillary blood samples. The FIA method is a rapid serological test that can be performed in the laboratory or used as POCT [48]. In our study, we focused on the sensitivity and specificity of the qualitative-quantitative detection of the IgG with the two methods compared, as previous studies have highlighted the risk of obtaining false positive results with tests for IgM, due to their potential cross-reactivity with common cold coronaviruses (such as HKU1, NL63, OC43, 229E) [49]. Indeed, in our study protocol, subjects who presented positivity for IgM antibodies underwent to oropharyngeal swab in order to verify the actual positivity to the SARS-CoV2. The latter modality is able to provide accurate results within 10 min with equivalent sensitivity and specificity, as confirmed by our data, both quantitatively and qualitatively, if compared to automated immunoassays. In particular, the results of our study suggest that, due to its easy implementation, the use of the FIA method might provide advantages when obtaining quick results is a key factor, i.e. the FIA test can prove useful in monitoring subjects that must be reintegrated into the workplace, ensuring workers health surveillance. In a wider perspective, this analytical method could be applied in different contexts such as facilities hosting communities, like assisted health residencies, convents, army barracks and prisons, for the purpose of applying a health surveillance model in epidemic areas.

A valid example of health surveillance model in epidemic areas was realized in the municipality of Vo, Padua (Italy) [50]. In this rural city, researchers performed a global screening of resident population that allowed accurate tracking of the viral transmission. In particular, this model should be applied both in subjects asymptomatic, potentially infectious, and in patients who have already manifested the disease. For this reason, the systematic use of health surveillance through a POCT might be a key factor in monitoring the epidemiological situation related to viral transmission, developing good socio-political strategies, with low cost, against the expansion of the epidemic. A further field of application of POCT, related to the detection of antibodies to SARS-CoV-2, could be that of sport. In fact, in some disciplines “social distancing” is not possible (for example football, rugby, martial arts etc.) therefore it is essential to evaluate positivity of athletes to SARS-CoV-2 [51, 52]. Therefore, this antibody screening could also be carried out to the public who goes to attend sport events or other mass events, such as concerts, public performances etc.

Further advantages of FIA ​​method are represented by simultaneous diagnosis of IgG and IgM in 10 min both on serum and on whole blood (by capillary sampling). Although it requires the presence of the operator during the entire analytical process (comparison with CLIA fully automated method), the FIA method in POCT, allows to concurrently carry out other biochemical assays, such as C Reactive Protein (CRP), troponin, procalcitonin [53, 54]. Moreover, the opportunity to perform the test outside clinical laboratories permits to reach larger groups of population without saturating the laboratories capacity. POCT may play an important role in large-scale testing to evaluate herd immunity for SARS-CoV-2. However, mistakes in the interpretation of results in situations that are not under the control of trained staff must be taken into consideration. For this reason, the development of automated reader devices could help to reduce human errors and increase sensitivity. In addition, such devices could support the communication of the screening results to a public health institutions to provide real-time information of seroprevalence in the population.

Finally, the FIA method also proves to be safer than oral swab sampling. In fact, the latter could cause sneezing and coughing, increasing the risks of operator exposure to the virus. The results of this study show a good reliability, in terms of sensibility and specificity, of POCT FIA method to check accurately the population screening for the antibodies SARS-CoV-2 research.

## Conclusions

FIA method could be helpful in assessing in short time, the possible contagiousness of subjects who, due to work needs, cannot guarantee “social distancing” to avoid the spread of COVID-19 by symptomatic and, above all by asymptomatic individuals. However, development of an automated FIA would ensure greater sensitivity associated with a relative decrease in the operator workload.

## Methods

### Design of the study and diagnostic methods

To assess the concordance between FIA and CLIA methods, a group of 100 subjects (49 males, 51 females, mean age 49,7 ± 4,5 years) have been selected to be tested with both techniques. The subjects were recruited from two different centers: the COVID Unit of the University Hospital Policlinico Tor Vergata (PTV), Rome, Italy and the Artemisia Lab-Alessandria (ALA), Rome, Italy. In each subject, blood samples were taken from antecubital vein, collected into vacutainer tubes and subsequently they were centrifuged and processed with both methods. In particular, we tested anti-Sars-CoV-2 antibody of all enrolled patients. Among these, 30 samples were collected from COVID-19 positive patients (determined by CLIA methods), belonging to Laboratory of Clinical Microbiology, University Hospital PTV, 30 COVID-19 negative samples (assessed by CLIA methods) were taken from ALA and subsequently all samples were re-analyzed in double blind with FIA method. In addition, 40 samples collected from subjects with COVID-19 suspected, were analyzed with both laboratory methods at ALA. To avoid biases of sampling methods, we performed the same sampling procedures in both diagnostic methods. The study protocol complied with the declaration of Helsinki was approved by the Ethical Committee of University Hospital PTV. All subjects were > 18 years and they all signed a full informed consent before the enrollment into the study. Exclusion criteria were: clinical conditions inducing immunosuppression such as neoplasms, solid or hematological, HIV and autoimmune diseases in the active phase and pregnancy.

The blood serum samples, collected into tubes contain spray-coated silica and a polymer gel for serum separation (Vacutainer, BD, Plymouth, UK), were used to perform the venous sampling. In order to guarantee operator safety, samples have been subjected to direct viral inactivation with dry heat, without preparing secondary aliquots, since this strategy has already proved an effective workload management [55].

Tubes were transported from the University Hospital PTV to the ALA in a container for biological material transport on dry ice. The samples analyzed in ALA first underwent a 37 °C dry-heat treatment, then were centrifuged for 20 min at 3500 rpm. Subsequently, anti-Sars-CoV-2 antibodies were analyzed with two methods: FIA method “AFIAS COVID-19 Ab- Boditech Med Inc.’s Technical Services” and CLIA method “SARS-CoV-2 Snibe Diagnostic” with the MAGLUMI instrumentation. Both the samples were processed sequentially with the two devices.

The first one is MAGLUMI™ 800 (New Industries Biomedical Engineering Co., Ltd. [Snibe], Shenzhen, China). It is an automated CLIA, featuring high throughput (up to 100 tests/h). According to the manufacturer’s inserts (271 SARS-CoV-2IgM, V2.0, 2020–03 and 272 SARS-CoV-2 IgG, V1.2, 2020–02), the SARS-CoV-2 IgM cut-off is 1.0 AU/mL, while the SARS-CoV-2 IgG cut-off is 1.1 AU/mL. Manufacturers claimed that the calculated clinical sensitivities of IgM and IgG were 78.65 and 91.21%, respectively, while specificities of IgM and IgG were 97.50 and 97.3%, respectively [10, 56].

The procedure of MAGLUMI test is performed in this sequence: the sample, buffer, magnetic microbeads coated with anti-human IgM or IgG monoclonal antibody are mixed thoroughly and incubated, forming immune-complexes. After precipitation in a magnetic field, the supernatant is removed and wash cycle is performed. Then SARS-CoV-2 recombinant antigen labeled with ABEI is added and incubate to form complexed. After precipitation in a magnetic field, the supernatant is removed, and then another wash cycle is performed. Subsequently, the Starter 1 + 2 are added to initiate a chemiluminescent reaction. The light signal is measured by a photomultiplier as relative light units (RLUs), which is proportional to the concentration of SARS-CoV-2 IgM present in the sample.

The AFIAS COVID-19 Ab sandwich immunoassay is a technique based on an automated fluorescent immunoassay system produced by Boditech Med Incorporated. This test uses a sandwich immunodetection method: fluorescence-labeled conjugates in a dried detection buffer binds to antibody in sample, forming antibody-antigen complexes, and migrates into nitrocellulose matrix to be captured by the other immobilized-anti-human IgG & anti-human IgM on test strip. The presence of antibodies in sample, forms the antigen-antibody complex and leads at an increase fluorescence signal on detector antigen, which is processed to show concentration of anti–SARS-CoV-2 IgG and IgM in sample respectively [22].

To ensure the correspondence between results obtained from capillary blood tests and those obtained from venous blood test with the FIA ​​method, both types of sampling were analyzed. Subsequently, the same sample was analyzed with the CLIA Snibe method with the automatic MAGLUMI tool.

To evaluate intra-series consistency and repeatability for both FIA and CLIA tests, 5-fold repeated test-retest was performed [24].

The production lots used to perform the tests described, were the following: Covid-19 FIA, AFIAS COVID-19 Ab. Boditech Med Inc.’s Technical Services Lot WHQDA12G EX 2021/12/16; Covid-19 IgM-CLIA MAGLUMI SARS-CoV-2 Snibe Diagnostic Lot 271,200,501 Ex2021/03/17; Covid-19 IgG-CLIA MAGLUMI SARS-CoV-2 Snibe Diagnostic, lot 2,722,000,501 Ex 2021/03/17.

### Statistical analysis

For both IgM and IgG analysis, we conducted two classes of analysis. Specificity, sensitivity and accuracy of the Covid-19 FIA (prediction set) were evaluated with respect to Covid-19 CLIA (ground truth). The sensitivity is the proportion of positive cases in COVID-19 FIA test out of the number of cases, which were positive in the COVID-19 CLIA test. Conversely, the specificity is the proportion of negative cases in COVID-19 FIA test out of the number of cases, which were negative in the COVID-19 CLIA test. Accuracy is the sum of true positive and the true negative in Covid-19 FIA test over the total cases. The accuracy’s 95% confidence interval (CI) were also calculated. Further, McNemar’s test was performed to test whether the row and column marginal frequencies are equal ― i.e., if the COVID-19 FIA results and the COVID-19 CLIA results significantly disagree one with each other. Statistical analysis was conducted using R software [57] and caret software libraries [58].

## Data Availability

All data generated or analyzed during this study are included in this published article.

## Abbreviations

ACE2: Angiotensin-converting enzyme 2
ALA: Artemisia Lab-Alessandria
ARDS: Acute respiratory distress syndrome
CI: Confidence interval
CLIA: Automated chemiluminescent immunoassay
CNCDs: Chronic non-communicable diseases
COVID-19: Corona virus disease-2019
CRP: C reactive protein
FIA: Flow immunoassay
MERS-Cov: Middle east respiratory syndrome coronavirus
NPV: Negative predictive value
ORF: Open reading frames
POCT: Point of care test
PPV: Positive predictive value
PTV: Policlinico Tor Vergata
RBD: Receptor binding domain
RLUs: Relative light units
RT- PCR: Reverse transcriptase polymerase chain reaction
TMPRSS2: Type 2 transmembrane serine protease
SARS-CoV-2: Severe acute respiratory syndrome coronavirus 2
WHO: World Health Organization
